# Supplementary material for: Are carbon emissions trading and green financial instruments synergistic? -Comprehensive quantitative research based on content analysis
Source: PLoS One. 2024 Mar 7;19(3):e0298601. doi: 10.1371/journal.pone.0298601 (PMC10919601; doi:10.1371/journal.pone.0298601)
Supplement: S1 Data — (ZIP) [file pone.0298601.s001.zip › Data/地级市Z_F工作报告.zip!/╔┬╬≈/╤╙░▓╩╨/└┤╫╘╣1⁄2╓┌║┼í╛┬φ┐╦╩2╛▌═°í┐_2015.docx]

各位代表：

现在，我代表市人民政府向大会报告工作，请予审议，并请市政协委员和其他列席人员提出意见。

一、2014年工作回顾

过去一年，我们紧紧围绕市四届人大四次会议确定的目标任务，坚持以统筹城乡发展总揽工作全局，积极应对持续加大的经济下行压力，攻坚克难，综合施策，经济社会发展呈现平稳运行、稳中有进态势。完成生产总值1386.09亿元，增长6.2%；全社会固定资产投资1541.07亿元，增长16.7%；地方财政收入168.1亿元，增长8.2%；城镇居民人均可支配收入30588元，增长10.7%；农民人均纯收入9779元，增长12.7%。

紧扣关键环节精准发力，经济保持平稳增长。针对能源价格持续下降、工业增长乏力、企业投资缩减的困难局面，及时出台促进经济增长的11条意见和10项措施，全力支持企业扩大生产销售。生产原油1716.4万吨，增长1.5%；加工原油1016.1万吨，下降0.3%；生产天然气18亿立方米，增长63.6%；煤炭4346万吨，增长6.3%。第一产业增加值113.7亿元，增长5.5%。粮食产量78.7万吨，增长5.8%；苹果产量261.4万吨，增长7.1%；蔬菜产量113万吨，增长6.6%；新增规模养殖户441户。接待游客3145.5万人次，实现旅游综合收入171.8亿元，分别增长10.5%和13.1%。完成社会消费品零售总额193.2亿元，增长13.5%。

大力培育支柱产业，经济结构调整取得新进展。全力推进工业园区建设，入园企业达到300户，富县双翼石化煤焦油深加工、洛川亿条编织袋等项目建成投产，富县能源综合利用、华能电厂、大唐热电联产和一批天然气液化项目有序推进，园区工业增加值增速高于规上工业7.4个百分点。启动建设现代农业示范园区74个，建成30个；市级以上龙头企业达到102家，专业合作社达到2296个，创建家庭农场1405个。十大革命旧址保护提升和五大文化旅游园区加快建设，西北局纪念馆建成开放，文安驿文化园区一期主体完工，中国革命艺术家博物院、延安大剧院开工建设。出台加快现代服务业发展的意见，组建了延安能源集团和民营投资公司，新增“四上”企业215户、注册企业3663户、个体工商户14475户，第三产业和非公有制经济占GDP比重分别达到21.9%和 21.1%。

加快四级城镇体系建设，新型城镇化快速推进。新区一期岩土工程基本完成，连接新老城区的尹家沟、核桃树塔2条道路建成通车，杜家沟、杨家岭2条道路具备通行条件。市级行政中心、市民服务中心和3所学校主体封顶，医院、住房、供水供电、公园广场等项目加快实施。旧城改造顺利推进，杜公祠、体育场片区改造、南门坡征迁和宝塔山、清凉山居民下山基本完成，累计搬迁居民10万人。杨家岭、龙湾隧道建成通车，一些“卡脖子”路段正在有效疏通。深入开展“五城联创”活动，加强城市精细化管理，持续加大旅游环境综合整治力度，游客满意度在全国60个样板城市中综合排名第37位。县城承载服务功能明显增强。重点镇和新型农村社区建设初步成型，基础设施、公共服务和产业园区逐步完善。5万多农民进城落户，城镇化率达到55.82%。

狠抓生态环境和基础设施建设，发展保障能力不断增强。完成退耕还林57.6万亩，造林绿化121.5万亩。治沟造地10.6万亩。实施王窑水库环境综合治理工程，持续推进锅炉煤改气、汽车油改气，淘汰黄标车和老旧机动车1.96万辆，城区空气质量优良天数按新标准达到252天，居全省第一。全面完成节能减排任务，化学需氧量、氨氮、二氧化硫、氮氧化物分别削减1.47%、1%、0.53%和4.4%。深入开展美丽乡村建设，公路、铁路沿线脏乱差得到有效整治。国家发改委将我市列为首批全国生态文明先行示范市。新机场、延安至延川高速、黄延高速扩能工程进展顺利，南沟门水库下闸蓄水，黄河引水工程全线推进。建成一批输变电工程，启动了国家A类城市电网建设。

全力办好民生实事，城乡居民享受到更多改革发展成果。城镇新增就业1.94万人，城镇登记失业率3.67%。调整了医疗保险报销政策，实现基本医疗、大病保险及救助制度无缝对接。建立了城乡居民丧葬补助制度，提高了居民基础养老金和大学生见习补助标准，完善了高龄老人、独生子女家庭补助制度。为18.3万城乡低保对象发放低保金4.37亿元。推行居家养老、社区服务模式试点，新八一敬老院和一批县乡养老机构建成投用。全面推行城区中小学校际联盟、学区制管理和招生制度改革，择校和大班额问题得到有效缓解。完成城区3所中职学校整合。延安医疗集团实质性运行。市中医院迁建启动实施，与西交大一附院签订合作办医协议。建设扶贫避灾移民搬迁集中安置点129个，开工3.48万套，完工2.83万套。8.6万人实现脱贫。开工保障房6.3万套，建成1.97万套。灾后重建进展顺利，70%的房窑倒损户入住新居。

着力破除体制机制障碍，发展活力进一步释放。创新融资方式，项目融资实现新突破。光大银行、西安银行入驻我市，延安农商行筹建工作有序推进。新增小额贷款公司7家，累计达到42家。积极推进城乡建设用地增减挂钩指标市域流转，争取周转指标2291.9亩。建立了城乡统一的土地资源公共交易平台，收储土地4780亩，出让3955亩。流转家庭承包经营耕地44.1万亩。完成食品药品监督管理体制改革，户籍制度、镇村机构等改革积极推进。对外开放取得新成效，招商引资到位资金511.8亿元，增长24.6%。延安海关开关运行，外贸进出口总额增长21.2%。

深入推进社会治理创新，社会大局和谐稳定。积极排查矛盾纠纷，集中化解信访积案，进京上访和非正常上访分别下降51.8%和72.5%。加强道路交通、消防、煤矿、危化品、森林防火、食品药品等重点行业和领域安全整治，安全生产事故起数和死亡人数分别下降13.8%和7.1%。大力推进平安延安创建，坚持开展“黄赌毒”、“两抢一盗”等严打整治专项行动，社会治安满意度持续提升。深入开展“六五”普法，全民法治意识不断增强。

加强政府自身建设，行政效能进一步提升。认真落实群众路线教育实践活动整改措施，解决了一批事关群众利益的突出问题。再造行政审批流程，全面推行并联审批、网上审批、重大项目全程代办、集中办证等制度，清理取消审批事项146项，精简率达到39%。推行财政资金项目化管理和部门预算公开，修订了机关事业单位差旅、培训、会议、公务接待、政府采购等管理制度，“三公”经费下降15%。清理清退公务用车99辆、办公用房2.2万平方米。自觉接受人大、政协和社会各界监督，向市人大报告了财政预算执行、审计、政府债务等方面工作，与市政协专题研究讨论了交通物流服务业发展问题，向老干部、民主党派、工商联和无党派代表人士及时通报经济社会发展情况，办理人大代表建议117件、政协委员提案457件，办复率100%。建立市政府领导联系人大代表、政协委员和基层群众制度，组织开展了19次公民代表走进市政府活动。

各位代表，过去一年，面对严峻形势和困难局面，取得这样的成绩实属不易，这是市委坚强领导的结果，是市人大、市政协监督支持的结果，是全市上下团结奋斗的结果。在此，我代表市人民政府，向全市干部群众、驻延部队指战员、武警官兵和公安民警致以崇高敬意！向大力支持政府工作的各位代表、政协委员和社会各界人士表示衷心感谢！

在总结成绩的同时，我们也清醒看到我市发展面临的一些突出矛盾和问题：受能源产品产量、价格双下降和企业生产效益下滑、投资能力下降等因素影响，规上工业、GDP、固定资产投资增速等指标与预期目标还有一定差距；非能源工业、第三产业和非公有制经济在经济总量中比重较小；一些重大产业项目推进较慢，推动产业转型、实现经济增速止跌回升的任务艰巨；区域、城乡发展不平衡，城乡居民收入差距依然较大；政府行政效能和服务水平还需进一步改进。对此，我们将高度重视，下功夫研究解决。

二、新常态下政府工作的总体思路和预期目标

标就是，么想，想到了就可以办到了当前，宏观经济形势正在发生深刻变化，中央作出我国经济发展进入新常态的重大战略判断。省委、省政府对全省经济发展思路作出新的调整部署，要求我们充分认识、理性看待、积极适应、主动引领新常态。新常态下延安发展面临巨大压力和挑战，我们要充分认识完成今年目标任务的艰巨性，进一步做好应对严峻复杂形势的思想准备。延长集团、长庆油田两大企业进入稳产状态，依靠增加石油产量拉动经济增长的动力减弱，新的接续产业培育较慢，经济下行压力持续加大；受宏观形势压力传导，企业投资信心和能力有所下降，我市近几年与中省企业合作实施的一批重大产业项目推进困难加大，结构调整过程将会更长更艰难；随着石油价格下降，企业效益下滑，财政增收难度加大,刚性支出不断增加，收支矛盾日益凸显;受市场需求不旺、项目开工不足等因素影响，就业矛盾和社会稳定风险加大，对各级政府统筹谋划、科学决策、维护稳定等提出了新要求。

新常态下实现动力切换、结构优化、提质增效，我们也面临新的机遇。国家实施“一带一路”战略，继续推进西部大开发，加快实施陕甘宁革命老区振兴规划，省委、省政府全面推进丝绸之路经济带新起点建设，为我们融入区域协调发展、参与国际国内经济合作创造了有利条件；我市非能源工业、第三产业和非公有制经济发展具有很大潜力，为我们加快结构调整、产业转型，推动经济步入中高速增长、产业向中高端迈进、增长动力实现多元支撑提供了发展空间；随着近几年统筹城乡和改革开放深入推进，长期以来制约发展的资金、土地和城乡二元体制等问题正在逐步破解，各类市场主体快速成长，发展环境不断优化，干部作风明显转变，发展潜能加速释放，将为我们在新一轮竞争中赢得先机。特别是习近平总书记春节前回延安视察，访贫问苦，慰问群众，视察新区，听取汇报，对延安脱贫致富、加快发展作出重要指示，使我们倍感亲切，倍受鼓舞，倍增信心，为我们加快三个延安建设增添了巨大动力。

综合上述考虑，今年政府工作的总体思路是：认真贯彻落实十八届三中四中全会、中央经济工作会议、习近平总书记系列重要讲话精神和省委十二届六次全会、市委四届七次全会的部署要求，坚持以统筹城乡发展为总揽，围绕稳中求进总目标，积极适应和引领经济发展新常态，继续加大稳增长、调结构、促改革、惠民生、防风险工作力度，全力保持经济平稳增长，推动产业转型实现新突破，城镇建设迈出新步伐，民生保障得到新提升，生态建设取得新成效。主要预期目标是：生产总值增长6%左右，全社会固定资产投资增长16%，地方财政收入按新口径同比增长12%，城镇居民人均可支配收入和农民人均纯收入分别增长11%和12%。

今年是“十二五”收官之年，也是我市加快统筹城乡、推动转型发展的重要一年。将今年经济增长速度定为6%左右，是综合分析各方面形势和要求，结合我市实际确定的。尽管这个目标低于全省水平，但对我们来讲，仍是一个需要付出艰辛努力才能实现的目标。这既是支撑我市自身发展、保障民生、完成“十二五”目标的需要，也是适应全省中高速增长、树立延安形象、凝聚发展信心的需要。我们要运用系统化思维做好各项工作，努力推动经济社会在新常态下实现新发展。坚持稳定骨干产业与发展新支柱产业并举，加快经济结构调整，积极培育新的经济增长点，着力构建具有延安特色的现代产业体系；坚持推进四级城镇体系建设，不断提升以人为核心的城镇化水平，全面加快城乡一体化步伐；坚持深化改革扩大开放，以重点领域和关键环节的改革带动整体工作推进，以招商引资和对外合作的新成效提升经济外向度水平，进一步激发内生动力和活力；坚持加强生态环境建设，不断巩固退耕还林成果，促进可持续发展；坚持保障和改善民生,按照兜住底线、完善制度、推进普惠的要求，实施好民生工程，使全市人民生活得更加自尊自信。只要我们坚定信心，迎难而上，积极作为，狠抓落实，就一定能够完成今年各项目标任务，实现“十二五”圆满收官，向省委、省政府和全市人民交出满意答卷。

三、2015年主要工作任务

（一）稳定骨干产业与培育新支柱产业同步发力，全力稳增长调结构促转型

保持能源工业稳定增长。支持延长集团实施科技兴油提高产能，启动永坪炼油厂扩能改造，加快成品油销售终端建设；力争长庆油田稳定产量，留地方加工量200万吨以上，完成1708万吨原油生产和1100万吨原油加工计划。加快陕煤集团3个矿井合并改造，建成富县党家河、芦村煤矿，开工车村一号煤矿，落实好煤炭外运绿色通道政策，力争煤炭产量达到5000万吨。加快天然气开发和转化利用，力争建成吴起、志丹、延川、安塞等县天然气液化项目，天然气产量达到40亿立方米，液化天然气产量达到70万吨。加快推进富县能源综合利用、洛川轻烃综合利用、延长油田伴生气资源综合利用等项目，开工大唐热电联产、华能电厂，加快黄陵煤制乙二醇、延川煤炭分质清洁利用等项目前期工作，力争年内开工建设。跟进落实中石油、中石化在延川、富县拟建项目，力争年内有实质性进展。

促进非能源工业快速发展。加快完善“三区九园”和县域工业园区水电路气、仓储、物流等基础设施和公共服务设施，加大项目实施力度，发展油田服务、装备制造、农产品加工、新型建材等产业，新增非能源规上企业20户。积极争取卷烟增产计划指标，优化产品结构，促进产量产值双提升。建成吴起50兆瓦、黄龙50兆瓦风电项目，开工延长50兆瓦风电、吴起50兆瓦光伏等项目，促进黄陵煤矸石电厂并网发电，全年发电量达到30亿千瓦时。加快宝塔、志丹陶瓷工业园区建设，开工志丹加压混凝土蒸气砌块、道路砖等项目。完成经开区孵化基地和深圳轻工产业园纺织服装、光纤通信等项目主体建设，开工安塞石油钻具加工维修、钢化玻璃等项目。非能源工业增速达到10%以上。

推动文化旅游和现代服务业突破发展。以打造全国红色旅游首选地为目标，抓好26个重大文化旅游项目建设，推进宝塔山、枣园、杨家岭和革命纪念馆整合创建5A级景区，加快文安驿、安定、铁边城等文化旅游名镇建设。加大旅游宣传培训和推介力度，开发网上旅游服务平台，持续开展旅游环境综合整治，稳步提升游客满意度，促进旅游人数和综合收入持续增长。加快构建能源装备、建材家居、农产品、小商品四大物流体系，推进安塞、吴起、富县、宝塔区等一批物流园区建设，启动新区大型市场综合体建设，支持一批物流企业做大做强。加快电子商务创业园区、电子商务服务平台建设，促进苹果、小杂粮等本地产品网上销售。规范家政服务标准和市场管理，扶持发展家政服务龙头企业。积极培育文化、健身、休闲等新兴服务业，鼓励社会资本兴办文化、体育经营服务企业。继续抓好“四上”企业培育和外来企业本地注册、商场市场统一结算，推进重点镇、新型农村社区连锁超市和农家店建设。全社会消费品零售总额增长13%，第三产业增加值增长9%。

加快发展非公有制经济。实施非公有制经济提升行动，继续扶持组建一批民营经济联合体和实业集团，鼓励进入能源转化、高新技术、新能源、新材料、节能环保、金融、公用事业等领域。支持民营企业加强与高校、科研机构合作，组建一批产业技术创新联盟、工程技术研究中心和重点实验室，促进科技成果转化利用，提高民营企业技术创新和市场竞争力。完善中小企业服务体系，培育一批中小企业特约服务机构，搭建企、地、银交流合作平台，解决中小企业融资难题。继续深化工商登记制度改革，简化审批程序，推行“三证合一”，稳步推进“个转企”，新增小微企业3000户。非公有制经济增加值占GDP比重达到22%以上。

大力发展现代农业。围绕重点镇和新型农村社区，继续加快现代农业示范园区建设，新启动13个，累计建成40个。稳步扩大特色农产品基地规模，粮食面积稳定在300万亩，继续推进苹果北扩，新增面积10万亩，新增设施蔬菜2万亩、露地菜1万亩，建成一批规模化养殖小区。积极引进推广新技术、新品种，提高农业设施装备、科技应用、生产经营水平，促进农业提质增效。积极培育大型龙头企业，提升专业合作社产业化服务水平，大力发展家庭农场、加工养殖大户和农村服务业，推进农村一二三产业融合发展。继续做大洛川苹果、延川红枣、延安小米、延安地椒羊四大特色品牌，完善品牌技术标准和管理办法，以品牌优势扩大市场占有率。完善社会化服务体系，强化农业生产经营服务和防灾减灾，支持电商、物流、商贸、金融等企业参与涉农电子商务平台建设，持续增加农民收入。

（二）抓好重大项目建设，保持固定资产投资较快增长

狠抓项目推进实施。继续完善项目清单管理、动态监管、企地定期会商等机制，全力抓好重大产业项目建设。加快推进交通、水利、电力等重大基础设施项目，建成延安至延川高速公路，加快黄延高速扩能工程、沿黄公路等项目建设，开工北过境线改建、姚店至市区快速干道、蒙华铁路延安段等项目，支持洛川、宜川、富县、安塞等县城过境路建设，开工吴起至定边高速公路，积极推进西包客运专线前期工作。完成延安新机场土方工程，启动连接道路、部分地面设施建设。加快黄河引水、南沟门水利枢纽工程建设，确保明年实现供水。继续推进气化延安、国家A类城市电网建设，建成一批输变电项目，气化一批重点镇和新型农村社区。

加大招商引资力度。主动融入丝绸之路经济带，加强与周边地区交流合作，扩大对外开放。围绕陶瓷产业、精细化工、生产性服务基地、农产品加工、非能源项目等五大重点领域，加强与央企、省企和省内外民企对接，积极承接产业转移，提高招商引资实效。落实重大招商项目领导包抓责任，加强招商队伍建设，强化招商项目跟进服务考核，推动项目落地建设取得新突破。招商引资到位资金增长20%。外贸进出口总额增长10%。

围绕“十三五”规划编制加强项目谋划争取。把握国家投资导向和产业政策方向，明确我市“十三五”发展目标、产业布局、重点任务。围绕国家实施“一带一路”战略、陕甘宁革命老区振兴规划和中央扶贫开发工作的新部署，积极谋划一批支撑长远发展的重大项目，精心策划包装，分年度推进实施。完善项目对口争取机制，打好延安牌，力争有更多重大项目列入中省规划。

（三）以人的城镇化为目标，继续推进四级城镇体系建设

全面加快新区建设。坚定不移实施“中疏外扩、上山建城”战略，加快新区基础设施建设，使新区初具现代城市功能和形象。建成5条新老城区连接道路和一批市政道路，实现新老城区畅通连接。加快水电气热管网和垃圾污水处理、中水回用等设施建设。建成市级行政中心、市民服务中心。3所学校秋季实现招生。加快政法系统业务用房、医院、干部职工住房、保障房和石油博物馆、历史博物馆、城市规划展览馆等重点项目建设。实施二期岩土工程，加快一期土地出让。加大招商引资力度，大力发展商贸、金融、文化、信息等服务产业，打造城市经济增长新引擎。

继续推进旧城改造。围绕“中国革命圣地、历史文化名城、优秀旅游城市”定位，持续推进三大片区改造，疏解老城密度，保护革命旧址，优化城市环境，提升文化内涵。加快三山绿化、山体公园、延河南河水景观和南门广场、凤凰山广场等项目建设，完成一批雕塑、小品建设和建筑物外立面改造。实施宝塔山至枣园沿线视线走廊综合治理，启动延河两岸、解放剧院广场改造和师范路、北桥沟、市场沟河滨公园等项目。继续推进棚户区改造，基本完成城市核心区域居民下山。统筹实施道路、电力、给排水、供热、通信等市政设施建设，不断完善服务承载功能。

推行城市精细化管理。加快国家智慧城市试点建设，完善城市管理监督指挥体系，实现全方位覆盖、全时段监控、网格化管理。深入推进“五城联创”，坚持开展市容秩序、环境卫生、户外广告、建筑渣土、占道经营等专项整治，实施市民文明素质提升行动。严格落实城市规划和公共空间管理等制度，划定城市开发边界，夯实市区镇村四级监管责任，持续开展市区和城乡结合部违法建设整治活动。

完善县城、重点镇和新型农村社区承载服务功能。加快县城建设，改善环境，彰显文化，提升品位，提高承载人口、聚集产业能力，打造一批宜居宜业的小城市。继续完善重点镇、新型农村社区基础设施和公共服务设施，加强产业园区配套，完成省市级重点镇主要建设任务，加快县级重点镇和新型农村社区建设，促进农村人口就地就近聚集。

（四）坚持兜底线保基本可持续，不断提升民生保障水平

千方百计扩大就业。以高校毕业生、城镇就业困难人员、农村转移劳动力等为重点，进一步完善就业扶持措施，努力扩大就业。设立创业扶持基金，提高小额贷款额度，加快大学生创业园和小企业创业基地建设。整合培训资源，加强就业培训，提高劳动者就业创业能力。加快发展小微企业，围绕完善城镇和社区管理服务增加更多就业岗位。城镇新增就业1.9万人以上，城镇登记失业率控制在4%以内，农村劳动力转移就业30万人。

持续提升社会保障水平。完善城乡居民养老保险一体化和医疗保险一体化制度，实现养老保险无障碍转移、医疗保险报销及时便捷。继续扩大工伤、生育、失业保险覆盖面，推进生育保险市级统筹。健全退休人员基本养老金和最低工资标准正常调整机制，促进社会救助与社会保障制度有机衔接。加大城乡低保对象核查力度，切实做到应保尽保。大力推行居家养老、社区服务模式，鼓励社会力量参与养老机构、残疾人托养机构建设，坚持为残疾人办好十件实事。

努力办好人民满意的教育。强化学校管理、校长教师培训和教育教学视导工作，深化教育教学改革，全面推广延安中学、宜川中学素质教育经验，提高全市中小学办学水平。充分利用革命历史文化资源，加强中小学生思想道德教育。巩固提升城区教育资源整合成果，继续推进校际联盟和学区制管理，严格落实义务教育就近入学政策。启动市实验中学、职业技术学院附中和宝塔区四中改制，支持宜川中学建设区域性重点中学。加快重点镇以上学校标准化建设和薄弱学校改造，促进义务教育均衡发展。从群众实际需求出发，适当恢复一些农村教学点。实施好第二期学前教育三年行动计划，逐步解决公办幼儿园比例偏低问题。加快普及青少年校园足球运动。重视发展特殊教育。办好延安职业技术学院，继续支持中国延安干部学院和延安大学加快发展。

提高公共卫生服务能力。继续推进优质医疗资源下沉，完善延安医疗集团对3所县医院一体化管理，启动对市第二人民医院、宜川县医院托管。发挥延安大学医学院和医疗集团总医院作用，加强基层医护人员培养，建立医疗人员双向流动机制。鼓励市县级医院创建重点学科，支持市医院创建三甲医院。完成市中医院迁建主体工程，加快市精神病医院和重点镇中心卫生院、村卫生室标准化建设，支持民间资本投资建设市妇女儿童医院和口腔专科医院。实施好居民健康教育工程，完善居民健康档案信息网络，加大孕产妇、先天性心脏病儿童等人群筛查和诊治力度，继续做好45岁以上居民体检、免疫接种等工作，提高群众预防保健水平。坚持计划生育基本国策，稳定低生育水平，促进人口长期均衡发展。

加大扶贫开发力度。认真落实陕甘宁革命老区脱贫致富座谈会精神，加大扶贫投入，加快社会事业发展，改善基础设施条件，提升公共服务水平。加大产业培育力度，加强贫困人口技能培训，增强造血功能和自我发展能力。继续推进千名干部包村联户扶贫工作，夯实工作任务和措施，实施精准扶贫，提高扶贫实效。加大项目资金整合捆绑力度，加快扶贫避灾移民搬迁和配套产业园区建设，确保70%以上群众进入城镇和社区安置。今年实施5万户、17.5万人搬迁。加强扶贫专项基金管理和分配，加快贫困群众脱贫步伐。今年实现7.84万人脱贫。

加快改善群众住房条件。新开工保障房3.2万套。启动老旧居民小区改造，加快农民工公寓建设和工矿企业棚户区改造。坚持公平公正公开，准确落实保障房对象。坚持租售并举，逐步推行共有产权和廉租房、公租房并轨运行，切实提高入住率。强化物业服务行业监管，提高物业服务管理水平。加快灾后住房重建和公共配套设施建设，确保入冬前房窑倒损群众全部入住新居。

丰富群众精神文化生活。加快市区、县城健身场所和重点镇、社区综合文体中心、健身广场建设，提升公共文化体育服务能力。继续推进陕北文化生态保护实验区建设，启动非物质文化遗产保护工程。鼓励创作文艺精品，坚持开展送戏下乡、文艺调演、体育赛事、全民健身等活动，积极配合中省做好第11届中国文化艺术节筹备工作。加大“黑网吧”、印制传播非法刊物和音像制品整治力度，净化文化市场环境。

着力保障群众饮食用药安全。健全食品药品和农产品安全监管协调联动机制，完善电子监管追溯体系。继续开展食品安全示范店、示范街和放心县创建活动，推进食品源头、生产加工、配送流通、餐饮消费、卫生环境、从业人员“六放心”工程建设。加快市区、县城豆制品、熟食品等加工园区建设，建成一批城市居民小区蔬菜直销店、直通车、生鲜超市和镇村放心粮油经销店。坚持抓好食品药品农产品安全监管和专项整治，保障群众饮食用药安全。

切实解决群众吃水难问题。加快推进节水型城市建设，逐步实施城市供水双管网改造，实行分类供水。建立雨水、污水收集系统，启动市区中水回用工程。逐步推行阶梯水价，促进节约用水。加快实施王窑水库综合治理，完成库区绿化、杏子河拦污闸坝建设和红庄水库防渗处理工程。启动子长红石峁水库建设，建成洛川、吴起、黄陵、黄龙、宜川、子长、富县7个县城和4个重点镇供水工程，解决13万农村人口安全饮水问题。

下功夫缓解城市交通拥堵。继续实施一批城市道路、隧道、桥梁和公交港湾、过街设施等项目，加快打通“卡脖子”路段和断头路，完善城市路网。逐步恢复市区改变用途的停车设施，建成一批新的停车场、停车楼，启动智能化停车和引导系统建设，推进公共停车设施市场化管理。搬迁市车管所、汽车客运东站，减轻城市交通压力。坚持公交优先原则，大力发展公共交通，及时调整运营线路和站点，更新运营车辆，规范管理，提升服务，方便群众出行。

（五）激活市场释放动力，全面深化各项改革

加强财税收支管理。制定国有资本经营收益征缴政策和非税收入激励措施，加强税收征管，保持财政收入稳定增长。实施全口径预算管理，继续推进专项资金项目化管理和绩效评价。调整和优化支出结构，压缩“三公”经费支出和财政项目投资，完成公车改革任务。推行“零基预算”，取消一切不必要支出。集中市县部分财力和石油行业新增资源税，建立全市保基本稳定调节基金。完善县域间对口帮扶机制，提高帮扶标准。强化新区建设、旧城改造土地经营收益监管，促进自求平衡、滚动发展。制定市级行政事业单位资产运营方案，推进房地产等政府资产盘活变现。

推动金融服务创新。整合投融资平台，培育集投资、担保、融资等功能的综合型投融资主体。继续支持发展小额贷款公司、村镇银行，加快推动农村信用社改制组建农村商业银行，鼓励社会资本创建地方民营银行。积极开展证券、保险、基金业务合作，引导符合条件的企业发行融资券和票据。探索建立农村土地经营权、宅基地使用权、农田经济林权等产权抵押融资机制，推进农民资产资本化，解决“三农”融资难问题。支持扩大政策性农业保险试点范围。加强金融市场监管，严厉打击非法集资活动，引导社会资本流向，规范金融市场秩序。

严格人事制度管理。加强市县政府机构、编制、人员管理，进一步规范机构、编制设置。健全完善公务员公开招录、遴选、事业单位凡进必考、临聘人员劳务派遣等制度。支持新区、经开区创新人事管理制度。按照中省部署推进机关事业单位养老制度改革，完善机关事业单位工资收入分配制度，实施县以下机关公务员职务与职级并行制度。推进职称制度改革，完善专业技能人才评价机制。

建立城乡一体的户籍制度。加快户籍制度改革，推行居住证制度，分类推进农业转移人口在城镇落户，促进进城农民在社会保障、就业培训、子女上学、公共卫生、社区养老、保障房政策、便民服务等方面享受与城镇居民同等待遇，提高户籍人口城镇化水平。

全面加强用地管理。集约节约利用土地，严格保护耕地，严肃查处违法用地行为。大力推进城乡建设用地增减挂钩指标市域流转，启动农村集体经营性建设用地流转入市试点改革。改革土地报批程序，提高土地审批效率。加大土地收储出让力度，提高土地利用效益。开展闲置土地专项清理，盘活土地存量资源。

加快农村土地确权和流转。加快农村土地承包经营权确权登记颁证工作，扎实做好档案资料清理、实地丈量勘查等工作，上半年完成权属核实确认和登记颁证。大力推进农村土地经营权流转，促进土地资本化运营、市场化配置。

推进市县政府和镇村综合改革。完成市县政府机构和撤乡并镇改革，积极稳妥推进行政村调整合并。强化乡镇综合职能，创新基层管理服务，建设功能完善、服务高效的镇村机构。

深化国有企业改革。推进市属企业分类管理，实行经营性国有资产集中统一监管。严格国有企业人事管理，全面推行聘任制。落实国有企业负责人薪酬管理办法，建立职工托管中心，维护职工合法权益。鼓励国有企业与民营企业相互参股控股、资产收购和兼并重组，加快发展混合所有制经济。

（六）持续改善生态环境，加快建设生态延安

深入推进生态建设。认真实施主体功能区规划，划定生态保护红线，严格控制开发强度。启动全国生态文明先行示范市建设，继续推进国家森林城市创建，坚持抓好退耕还林、天然林保护、三北防护林等林业重点工程，完成造林50万亩。扎实推进公路、铁路、河流沿线森林廊道建设，继续扩大城镇和新型农村社区人均绿化面积。启动南泥湾国家湿地公园、经开区湿地公园建设。完成治沟造地10万亩。加快实施延河综合治理，完成中心城区水环境整治工程。编制洛河治理规划，积极推进洛河治理。

加强重点领域污染治理。围绕创建国家环保模范城市和开展国家低碳城市试点，加强环保执法和应急能力建设，坚决防止重特大污染事故发生。加大治污降霾力度，推进城市燃煤供应与监管职能分离，加快实施锅炉煤改气、汽车油改气，加强建设工地扬尘治理。提高污水回收和处理能力，强化油区环境综合治理和输油管线安全管理。加强农村面源污染治理，继续开展生态乡村创建活动，保护蓝天白云、清水净土。

强化节能减排。完善建设项目节能评估和环境影响评价机制，健全节能减排指标监测和考核体系。加强重点用能单位节能管理，强化节能指标约束。积极推广石油、煤炭节能环保技术和工业、建筑、交通等领域节能产品。单位生产总值能耗下降3.3%，完成主要污染物减排任务。

持续推进美丽乡村建设。继续抓好重点镇、社区和公路、铁路沿线环境综合整治，统筹实施道路硬化、垃圾污水处理、河道整治、厕所改造、绿化美化等工程，完善乡村公共服务设施。在乡镇、社区、中心村实行规划管理，推进房屋建设、公共基础设施、产业配套统一规划实施。

（七）创新社会治理方式，保持社会大局和谐稳定

积极推进社会管理创新。推行“一联三化”和“两说一联”服务机制，加强一站式便民服务中心建设，完善社区工作标准体系和评价制度，健全基层社会管理服务体系。鼓励和支持社会力量参与社会治理，推动政府向社会购买公共服务。关心农村空巢老人、留守妇女和儿童，实现城乡人口管理一体化和服务全覆盖。

努力化解社会矛盾。坚持“三官一律”进社区、社区民警专职化、网格化管理，加强社情民意、网络舆情的引导和处置，认真解决土地征用、房屋拆迁、企业改制、环境污染等方面突出问题。完善信访、调解、复议、诉讼协调互动机制，畅通群众诉求表达和权益保障渠道。加强企业信用监管体系建设。做好民族宗教事务管理。深入推进平安延安建设，完善立体化社会治安防控体系，提高社会面管控水平，努力维护社会和谐稳定。

强化安全生产监管。完善综合监管、行业监管、属地管理和企业主体“四位一体”安全责任体系，坚持抓好道路交通、建筑施工、消防等重点领域和特种行业安全监管，落实挂牌督办、警示约谈、黄牌警告、绩效考核和“一票否决”等制度，坚决遏制重特大事故发生。健全应急管理体系，完善重大突发事件处置预案，加强应急专业抢险队伍建设，提高应急处置能力。

四、全面加强政府自身建设

增强法治意识，依法履行政府职能。全面落实推进依法治国战略部署，加快建设职能科学、权责法定、公开公正、廉洁高效的法治政府。健全政府依法决策机制，把公众参与、专家论证、风险评估、合法性审查、集体讨论决定作为政府重大决策法定程序，确保决策程序正当、过程公开、责任明确。积极推进综合行政执法改革，落实行政执法人员持证上岗、资格管理制度和行政执法责任制。自觉接受人大及其常委会的法律监督、工作监督和人民政协的民主监督，认真办理人大代表、政协委员建议和提案，坚持向人大报告工作、与政协协商工作、向老干部、民主党派、工商联和无党派代表人士通报工作制度。

推进政务公开，让权力在阳光下运行。加快建立政府权力清单、责任清单、部门专项资金管理清单和企业投资项目负面清单制度，继续改革行政审批流程，进一步精简行政审批，全面清理非行政许可事项，提高办事服务效率。完善政务公开制度，拓展公开范围，重点推进财政预算、公共资源配置、重大项目实施、社会公益事业建设等领域信息公开，所有面向社会服务的部门都要依法公开办事服务信息。加强政府电子政务服务平台和市县政务中心标准化建设，积极推行政务服务“一网化”管理，尽快实现行政审批和便民服务事项一站式网上办理，全流程信息公示。做好新兴媒体的应对引导，主动接受社会监督和舆论监督。继续开展公民代表走进市政府活动，加强市民热线和网民留言办理。

持续转变作风，推动工作落实。大力弘扬延安精神，深入开展“三严三实”专题教育活动，着力解决“四风”突出问题，持之以恒抓好作风建设。加强调查研究，精简会议文件，狠抓工作落实。全面推行群众和服务对象监督评价部门及窗口单位、限时办结等制度，坚决整治门难进、脸难看、事难办和推诿扯皮、吃拿卡要等行为。增强责任和担当意识，保持奋发有为的精神状态，对懒政庸政怠政、慢作为不作为、不敢抓不敢管的干部，强化行政问责和效能监察，在全市政府系统形成勇于担当、勤勉尽责、善谋善为的良好氛围。

坚持从严治政，树立清正廉洁形象。继续抓好政府系统群众路线教育实践活动整改落实，进一步夯实政府系统党建和党风廉政建设主体责任，充分发挥党员干部模范带头作用。坚持用制度管权管事管人，对公共资金、国有资产、国有资源和领导干部履行经济责任情况实行审计全覆盖。严格落实八项规定，厉行勤俭节约，反对铺张浪费，加强领导干部执行公务接待、因公出国境、住房和办公用房、车辆配备、个人重大事项报告等相关制度的监督检查，严肃查处违规违纪行为，以实际行动赢得人民的信任和支持。

各位代表，我们面临的任务艰巨而繁重，肩负的责任重大而光荣。让我们以习近平总书记回延安、回陕西重要讲话精神为指引，在市委的坚强领导下，扎实苦干，奋发进取，努力推动经济社会在新常态下实现新发展，为圆满完成“十二五”规划目标、加快统筹城乡发展做出新的贡献！
